# Probiotics for treating novel coronavirus with diarrhea: A protocol for systematic review and meta analysis

**DOI:** 10.1097/MD.0000000000021617

**Published:** 2020-09-18

**Authors:** Min Liu, Hongqiu Zhu, Yaling He, Ying Zhu, Xiaodan Hu, Yanling Zeng

**Affiliations:** a The Affiliated Hospital of Chengdu University of Traditional Chinese Medicine; b Department of Gynaecology, School of Medical and Life Sciences, Chengdu University of Traditional Chinese Medicine/ Reproductive and Women-Children Hospital of Chengdu University of Traditional Chinese, Chengdu; c Southwest Medical University, Luzhou City, Sichuan Province, China.

**Keywords:** coronavirus disease 2019, diarrhea, probiotics, protocol, systematic

## Abstract

**Background:**

The study aims to evaluate the efficacy and safety of probiotic therapy for coronavirus disease 2019 with diarrhea.

**Methods:**

The following electronic bibliographic databases will be searched to identify relevant studies from December 2019 to December 2020: MEDLINE, PubMed, Embase, the Cochrane Central Register of Controlled Trials, China National Knowledge Infrastructure, Chinese Technical Periodicals, Wan-fang data, Chinese Biological Medicine Database, and other databases. The search results will not be restricted by language, all included articles were randomized controlled trial. Two independent researchers will conduct article retrieval, de-duplication, filtering, quality assessment, and data analysis through the Review Manager (V.5.3). Meta-analysis, subgroup analysis and/or descriptive analysis were performed on the included data.

**Results:**

High-quality synthesis and/or descriptive analysis of current evidence will be provided from outcomes.

**Conclusion:**

This study will provide the evidence of whether probiotics is an effective and safe intervention for coronavirus disease 2019 with diarrhea.

PROSPERO registration number: CRD42020192657.

## 1 Introduction

The emergence and spread of coronavirus disease 2019 (COVID-19) since December 2019, due to its high infectivity and widespread vulnerability among populations, has posed enormous challenges to the health and economic development of people around the world. As of 23 June 2020, more than 9.2 million confirmed cases and more than 476,000 deaths are reported globally. In Diagnosis and Treatment Plan for COVID-19 (trial version 7) issued by the National Health Commission of China, it is noted that transmission through respiratory droplets and close contact are the main means of transmission. Clinically, fever, fatigue, and dry cough are the main manifestations, and diarrhea is the first or accompanying symptom in some patients.^[1]^ Chen analyzed 99 patients with COVID-19 in Wuhan, and the results showed that most of them had fever or cough when they were admitted to hospital, but 2% had diarrhea and 1% had nausea and vomiting.^[2]^ Guan conducted a statistical analysis on 1,099 COVID-19 patients, and found that about 3.7% of them had diarrhea symptoms and 5.0% had vomiting.^[3]^ Wang reported 138 COVID-19 patients that 10.1% had diarrhea, while 16.7% of 36 patients admitted to the intensive care unit had diarrhea.^[4]^ In case reports of the United States and Vietnam, gastrointestinal symptoms such as diarrhea and nausea have been described.^[5,6]^

According to the literature, the intestinal tract is an important immune organ, which is home to a large microbial population that can affect the body's respiratory tract through immune regulation.^[7,8]^ COVID-19 can be considered a self-limiting disease, and the patient's own immunity plays an important role throughout COVID-19 disease. With diarrhea-based digestive symptoms, it will disrupt the steady state of intestinal microorganisms, affect the body's immune function, and accelerate the progression of COVID-19, so there is an urgent need to add to the treatment of the patient's intestinal immune system. Probiotics as an intestinal microbe regulator, not only can regulate the intestinal micro-ecology, but also strengthen the body's immune system, inhibit allergic reactions, and help the body to fight cancer and obesity, especially in the anti-viral immunomodulation of significant role.^[9,10]^ Therefore, in patients with COVID-19 diarrhea, the intestinal micro-eco-regulator, represented by probiotics, may be a therapeutic choice for clinicians. However, there is still a lack of evidence-based evidence to support probiotic treatment of patients with COVID-19 with diarrhea, so it is necessary to conduct further review and provide evidence to clinicians. This review, which starts with probiotics, aims to assess the effectiveness and safety of probiotic treatment of COVID-19 diarrhea.

## 2 Methods

### 2.1 Study registration

This systematic review protocol has been registered in the PROSPERO (CRD 42020192657). The protocol refers to the guide book of Preferred Reporting Items for Systematic Reviews and Meta-Analyses Protocols (PRISMA-P).^[11]^

### 2.2 Search strategy

MEDLINE, PubMed, Embase, the Cochrane Central Register of Controlled Trials, China National Knowledge Infrastructure, Chinese Technical Periodicals, Wan-fang data, and the Chinese Biological Medicine Database will be searched. The search results will not be restricted by language, but will be limited to human studies only. In addition, the reference lists of all identified articles will be examined to identify studies not captured by electronic searches. The key search terms are ([“novel coronavirus” OR “new coronavirus” OR “2019 nCoV” OR “COVID-19” OR “2019 novel coronavirus” OR “Coronavirus disease 2019”] AND [“Probiotics” OR “live biotherapeutics”] AND [“diarrhea”] AND [“randomized” OR “randomly” OR “randomized controlled trial” OR “clinical trial”]). These search terms are shown in Table 1. Combinations of Medical Subject Headings (MeSH) and text words will be used. The search strategy will follow the Preferred Reporting Items for Systematic reviews and Meta-analysis (PRISMA) guidelines.

**Table 1:**
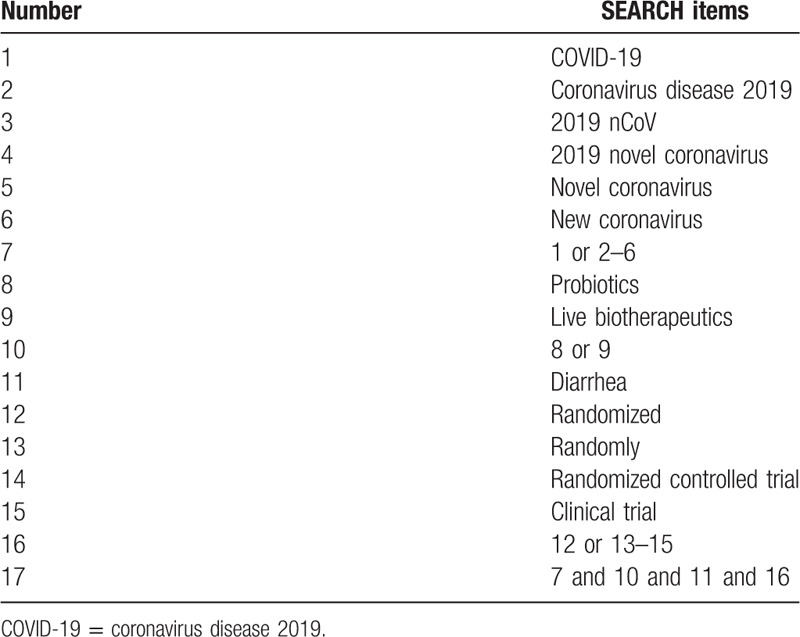
Search strategy for the PubMed database.

### 2.3 Study selection

#### 2.3.1 Type of study

We will include articles correlated to probiotics for treating COVID-19 patients with diarrhea. Due to language limitations, we searched Chinese and English articles to obtain more true and objective evaluation. All included articles were randomized controlled trial. If an experiment does not explain randomization, the article will be considered as high risk in random sequence generation.

#### 2.3.2 Types of participant

COVID-19 with diarrhea symptoms in patients over 18 years old will be included, without limits on gender, nationality, race, and disease classification.

#### 2.3.3 Type of intervention

Probiotics or probiotics combined with other routine treatments. We will compare the following interventions: other routine treatments such as Chinese medicine, acupuncture, antibacterial or antiviral drugs, and so on.

#### 2.3.4 Types of outcomes

Primary outcomes were the diarrhea symptom score at the end of treatment and at the end of follow-up, the rate of conversion of common types to severe types, and the times to, and rates of patients becoming negative for COVID-19. Secondary outcomes were symptom score (based on fever, fatigue, cough, difficulty in breathing, poor appetite, and so on), pulmonary function, results of chest computerized tomography, serum cytokine levels, days to the disappearance of fever, length of stay in hospital, the use (including the dosage and duration of administration) of corticosteroids, quality of life, and adverse events.

### 2.4 Data collection

#### 2.4.1 Data management

Endnote X9.1 will be used to manage search results and filter them. Statistical calculation will be performed by RevMan5.3 software and sensitivity analysis will be performed by Stata/SE 15.1 software.

#### 2.4.2 Data extraction

**Figure 1:**
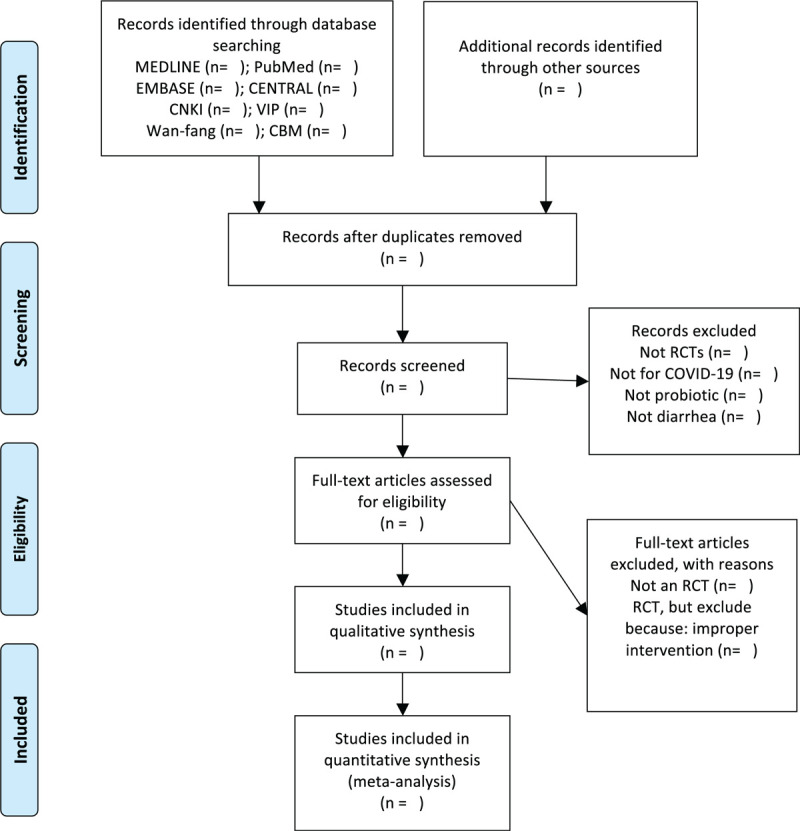
PRISMA flow diagram of the study process.

PRISMA flowchart was selected to show the literature selection process of the whole study (Fig. 1). Two authors will screen the studies retrieved during the searches against the inclusion and exclusion criteria, and those meeting these criteria will be selected for inclusion. Two authors will independently use data collection tables to extract the following data from the selected studies: the characteristics of the study (first author, title, publication year, study design), participants (sample size, age, sex, stage, and severity of disease, comorbidity), intervention (duration, frequency, types of probiotics, types of control group), pneumonia-related outcomes (all outcomes, main conclusions), adverse reactions, and other information. If necessary, we may also contact the original author of a given study to provide additional relevant information. The results will be cross-checked by the 2 authors, and any differences will be settled through negotiation, with any continuing differences of opinion being arbitrated by a third author.

#### 2.4.3 Risk of bias assessment

For all included studies, 2 authors will independently assess the risk of bias using the Cochrane risk of bias tool. The risk of bias for the studies will be divided into 3 levels (low risk, high risk, and unclear) based on the following domains: sequence generation, allocation concealment, blinding of outcome assessors and participants, incomplete outcome data, selective outcome reporting, and other sources of bias. Any discrepancies will be resolved by discussion with a third author. We will attempt to clarify unclear or inadequate content by contacting the corresponding author.

#### 2.4.4 Dealing with missing data

We will do our best to ensure the integrity of data. Due to the possibility of data loss in the literature, we will contact the corresponding author by email or other means. If the missing data is not available, the study will be excluded from the analysis.

### 2.5 Statistical analysis

#### 2.5.1 Assessment of reporting bias

In this analysis, publication bias and other reporting bias will be assessed by creating funnel plots.

#### 2.5.2 Data synthesis

Data consolidation will be conducted using RevMan software (version 5.3). Continuous data will be represented as the mean difference (MD) with 95% confidence interval (CI) between the 2 groups, while dichotomous data will be represented as the relative risk (RR) with 95% CI. The heterogeneity between the studies will be assessed using the *Q* test and the *I*^2^ test. If the value of *I*^2^ test is less than 50%, the fixed effect model will be used for data synthesis, whilst if the *I*^2^ test result is between 50% and 75%, the random effects model will be used for the data synthesis. If the *I*^2^ test is higher than 75%, we will look for the possible causes of the heterogeneity from clinical and methodological perspectives, and will provide a descriptive analysis or subgroup analyses.

#### 2.5.3 Subgroup analysis

A subgroup analysis will be performed in order to evaluate the different probiotics administered (the types of probiotics and the duration of the therapy), and the other possible factors that may lead to clinical heterogeneity, such as different probiotics and other combined therapies, different course times, different outcome indicators, and so on.

#### 2.5.4 Sensitivity analysis

If there is significant heterogeneity in trials included after subgroup analysis, sensitivity analysis will be performed to assist in the exploration of the source of heterogeneity. We will delete 1 study at a time, and then analyze other studies to estimate whether a single study would have a significant impact on the results.

#### 2.5.5 Grading the quality of evidence

This article will use the evidence quality assessment method to evaluate the results of the analysis. GRADE is generally applied to a large amount of evidence. It has 4 ratings: high, medium, low and very low. The score is used to assess deviations, inconsistencies, discontinuities and inaccuracies in test results.

#### 2.5.6 Ethics and dissemination

The content of this article does not involve moral approval or ethical review and will be presented in print or at relevant conferences.

## 3 Discussion

Covid-19 is highly contagious, has a variety of transmission routes, and is unpredictable in its development, seriously affecting social life and human health.^[1]^ Although the number of cures is increasing, there is no specific drug for the new coronavirus and is still in the treatment stage of antiviral diseases. However, long-term use of broad-spectrum antibiotics, glucocorticoids, stress and other conditions may cause intestinal microecological imbalance.^[12,13]^ Based on the role of probiotics in fighting respiratory virus infection and maintaining intestinal microecological balance,^[14]^ combined with the available evidence, probiotics can be used clinically as appropriate in patients with COVID-19 diarrhea who do not have contraindications for the use of probiotics, but there is no evidence-based evidence of safety and efficacy. The purpose of this review is to assess the benefits of probiotics for patients with COVID-19 diarrhea, with a view to providing alternative therapies for clinicians, as well as new ideas for the prevention and treatment of COVID-19.

## Author contributions

**Conceptualization:** Min Liu, Hongqiu Zhu, Yaling He.

**Investigation:** Min Liu, Ying Zhu, Xiaodan Hu.

**Methodology:** Min Liu, Hongqiu Zhu, Yaling He, Yanling Zeng.

**Project administration:** Min Liu, Hongqiu Zhu, Yaling He.

**Writing – original draft:** Min Liu, Yaling He.

**Writing – review & editing:** Min Liu, Hongqiu Zhu.

## Abbreviations

Abbreviation: COVID-19 = coronavirus disease 2019.
